# Editorial: Cellular and Molecular Mechanisms of Neurotrophin Function in the Nervous System

**DOI:** 10.3389/fncel.2020.00101

**Published:** 2020-04-28

**Authors:** Pedro Bekinschtein, Oliver von Bohlen und Halbach

**Affiliations:** ^1^Institute of Cognitive and Translational Neuroscience (INCYT) CONICET-Fundación INECO y Universidad Favaloro, Buenos Aires, Argentina; ^2^Institut für Anatomie und Zellbiologie, Universitätsmedizin Greifswald, Greifswald, Germany

**Keywords:** neurotrophic factors, BDNF (brain-derived neurotrophic factor), NGF (nerve growth factor), CNS—central nervous system, neuronal plasticity

The discovery of nerve growth factor (NGF) by Rita Levi-Montalcini in the 1950s represents an important milestone in the processes that led to modern cell biology (Aloe, 2004). NGF is a member of the neurotrophin family. Neurotrophins are a family of proteins that regulate development, maintenance, and function of vertebrate nervous systems. They serve as survival factors to ensure a match between the number of surviving neurons and the requirement for appropriate target innervation and also regulate cell fate decisions, axon growth, dendrite pruning, the patterning of innervation and the expression of proteins crucial for normal neuronal function, such as neurotransmitters and ion channels. They signal through specific tyrosine kinase receptor (trkA, trkB, trkC) and the low affinity receptor p75NTR. Moreover, the precursors of the neurotrophins (“pro-neurotrophins”) are discussed to be biologically active by signaling through specific receptors. Brain-derived neurotrophic factor (BDNF), NGF and the neurotrophins 3 and 4 (NT3, NT4) as well as their precursors (pro-neurotrophins) are not only expressed during development, but also in the postnatal brain.

Neurotrophins also have important functions in the mature nervous system. In particular, BDNF is involved in learning and memory and in neuronal plasticity. Changes in BDNF expression especially in the hippocampal formation are associated, among others, with psychiatric disease. BDNF levels are reduced in postmortem brain samples and in the blood of depressed patients, and these reductions are reversible by successful antidepressant treatment (Castren and Rantamaki, 2008). Moreover, in humans, the BDNF val66met polymorphism might represent a biological signature for the neuroanatomical and cognitive abnormalities commonly observed in patients suffering from bipolar disorders (Cao et al., 2016). Moreover, recent data indicate that BDNF concentrations were significantly lower in patients with attempted suicide/ideation and that therefore BDNF concentrations could serve as a response marker for antidepressant treatment in major depressive disorder (Ai et al., 2019).

Interventions like exercise or antidepressant treatment can enhance the expression of BDNF in the brain. In the review article by Miranda et al. the role of BDNF as a key molecule for memory processes in the healthy and the pathological brain is reviewed. While the role of BDNF in synaptic plasticity and in memory formation is well-known, its participation in memory reconsolidation has been less studied. In the Mini-review by Gonzalez et al. the involvement of BDNF signaling in memory reconsolidation is discussed.

The hippocampus is a key structure for spatial and declarative memory formation. Most of our knowledge on synaptic plasticity in mammals comes from hippocampal circuits, including the famous paradigm of long-term potentiation (von Bohlen und Halbach et al., 2018). Moreover, adult hippocampal neurogenesis is thought to be related to learning and memory functions. Interestingly, neurotrophins are involved in hippocampal long-term potentiation as well as in adult neurogenesis implicating an important role in neuronal plasticity (von Bohlen und Halbach and von Bohlen und Halbach, 2018). The Mini-review by De Vincenti et al. focuses on mechanisms that modulate and diversify BDNF functions and its implications for hippocampal synaptic plasticity, thereby shedding light to novel mechanisms that are discussed for the rapid, localized, and dynamic control of BDNF release and function. In addition to that Mini-review, the original article by Foltran et al. analyzes the differential hippocampal expression of BDNF isoforms and their receptors under diverse configurations of the serotonergic system in a mice model of increased neuronal survival. This work sheds light on the role of the different BDNF isoforms in the regulation of neurogenic process taking place in the murine hippocampal formation.

Activation of the low affinity receptor p75NTR is known to trigger synapse loss and neuronal death. These pathological features are also caused by the human immunodeficiency virus-1 (HIV) envelope protein gp120, which increases the levels of proBDNF. The research article by Speidell et al. portrays the interaction of p75NTR with the human immunodeficiency virus-1 (HIV) envelope protein gp120 in the brain and suggests that activation of p75NTR is one of the mechanisms crucial for the neurotoxic effect of gp120. Furthermore, the research article by Zhang et al. focuses on the involvement BDNF and NGF in peripheral nerve injury and indicates that increased levels of BDNF and NGF are associated with promoted sciatic nerve regeneration and improved nerve function.

Aside from the neurotrophins further substances have neurotrophic properties. Among them is the well-known family of fibroblast growth factors as well as the insulin like growth factors. The article by Chen W. et al. highlights the capacity of insulin-like growth factor-1, derived from astrocytes, to protect neurons against excitotoxicity. Recently, it has been described that interleukin 1 also plays a role in the nervous system and the article by Chen G. et al. entitled “Interleukin-1β promotes Schwann cells de-differentiation in Wallerian degeneration via the c-JUN/AP-1 pathway” indicates that interleukin 1ß plays an important role in Wallerian degeneration that is associated with alterations in p75NTR expression.

Together, the series of articles, should give a brief overview on the roles and function of neurotrophic substances in the nervous system. Since one of the best-analyzed systems in this context are represented by the neurotrophins and especially by BDNF. Thus, the mini reviews mainly focus on the actions of BDNF. However, since other factors also have been found to have neurotrophic properties or interact with the neurotrophin system, specific original articles were also included in the “Special Issue.” We hope that this selection of articles will be helpful to get insight in the fascinating world of the neurotrophins and their actions in the central nervous system.

## Author Contributions

PB and OB contributed equally to the special issue and the editorial.

## Conflict of Interest

The authors declare that the research was conducted in the absence of any commercial or financial relationships that could be construed as a potential conflict of interest.
